# Oat β Glucan Ameliorates Renal Function and Gut Microbiota in Diabetic Rats

**DOI:** 10.3389/fnut.2022.875060

**Published:** 2022-05-09

**Authors:** Ruoyu Wang, Zhaofeng Zhang, Sumiya Aihemaitijiang, Chen Ye, Mairepaiti Halimulati, Xiaojie Huang, Haoyuan Qin

**Affiliations:** ^1^Department of Nutrition and Food Hygiene, School of Public Health, Peking University, Beijing, China; ^2^Beijing's Key Laboratory of Food Safety Toxicology Research and Evaluation, Beijing, China; ^3^Department of Nutrition and Food Studies, Steinhardt School, New York University, New York, NY, United States

**Keywords:** oat β glucan, diabetic nephropathy, gut microbiota, inflammation, renal function

## Abstract

Diabetic nephropathy is a severe complication of diabetes and the leading cause of end-stage renal disease and death. Therefore, we must find effective prevention and treatment approaches to the problem. Oat has a long history of use for its nutritional and medicinal properties, such as maintaining physiological blood glucose levels. Oat β glucan is one of the major bioactive substances produced by oat. However, the protective effects of oat β glucan on diabetic nephropathy are still unclear. This study generated a rat model of diabetic nephropathy to explore the potent therapeutic ability and mechanism of oat β glucan in renal function by 16S rRNA genes sequencing. Diabetic nephropathy model was established in forty rats by left nephrectomy and single intraperitoneal injection of streptozotocin. These rats were randomly divided into the model group and three oat β glucan intervention groups. Twenty rats underwent sham operation and were randomly divided into normal control group and oat β glucan control group. Animals were treated by oral gavage for 8 consecutive weeks. The results showed that oat β glucan reduced blood glucose level and improved renal function (*P* < 0.05). Oat β glucan significantly improved serum inflammatory levels (*P* < 0.05). The diversity of intestinal microflora in diabetic nephropathy rats decreased with time prolongation, while oat β-glucan reversed the result. Compared with the model group at week 8, the abundances of *Eubacterium, Butyricicoccus*, and *Ruminococcus* were elevated significantly after oat β glucan intervention (*P* < 0.05). Correlation analysis indicated that abundances of *Eubacterium, Butyricicoccus*, and *Ruminococcus* were significantly negatively correlated with the levels of renal impairment markers. In summary, the findings of this study showed that oat β glucan can increase the diversity of intestinal flora, regulate the composition of intestinal flora, modulate intestinal flora metabolism, alleviate the inflammatory response, and further delay the development of diabetic nephropathy. Therefore, oat β glucan has the potential to be developed into the novel and safe drug for diabetic nephropathy.

## Introduction

Diabetes mellitus is a group of metabolic diseases that are characterized by hyperglycemia and arise from defects in insulin secretion, insulin action, or both (1). In recent years, with the aging of the population and the change of people's lifestyles, the prevalence of diabetes is growing annually (2). The International Diabetes Federation estimated that, globally, the prevalence of diabetes is expected to exceed 625 million people by 2045 (3). Diabetic nephropathy (DN) is one of the most frequent and serious chronic complications of diabetes mellitus and supervenes as the result of microvascular lesions in the renal glomeruli (4). Four to seventeen percentage of patients with type 1 diabetes will develop DN 20–30 years after their initial diagnosis. Up to 20% of patients with type 2 diabetes already have DN when diagnosed with diabetes and a further 30% to 40% develop DN, mostly within 10 years of diagnosis (5). DN is considered the leading cause of end-stage renal diseases and increases the risk of death (6). It poses a huge social medical and economic burden to individuals, families and the country. Therefore, we must find effective prevention and treatment approaches to the problem.

The etiology and pathogenesis of DN are complex and diverse. Studies suggest that factors other than hyperglycemia, such as hypertension, dyslipidemia, oxidative stress, and inflammatory response, may play a role in DN pathogenesis (7). Pharmacologic treatments for DN are mainly hypoglycemic (such as sulfonylurea drugs, biguidine drugs, α-glycosidase inhibitors, and insulin) and hypotensor (such as angiotensin converting enzyme inhibitors, angiotensin receptor blockers, and calcium channel blockers). However, they often cause serious side effects such as low glucose levels, lactic acidosis, swelling of legs or ankles, and stomach discomfort (8). In non-pharmacological intervention aspect, a healthy lifestyle with regular physical activity and healthy eating is recommended. However, it exhibits limited clinical efficacy and fails to prevent the development of DN (9). Therefore, it is necessary to define pathogenesis of DN and find more effective measure at the source.

As is well-known, the gut microbiota is an intricate ecosystem in which interactions exist among microbes and between microbes and their host (10). It has been shown that gut dysbiotic-microbiota has been implicated in the development of DN (11). Based on the gut-kidney axis theory, dysbacteriosis disrupts the gut barrier and thus increases gut permeability. Consequently, influx of endotoxins and uraemic toxins into the kidney via the circulation stimulates the activation of inflammatory responses both locally and systemically. In turn, deterioration of kidney function leads to gut dysbiosis (12). Studies have revealed that chronic low-grade inflammation can aggravate renal cell apoptosis and fibrosis, leading to the development and progression of DN (13). Therefore, modulating the gut bacteria composition may be a potential effective therapy for DN.

Oat (Avena sativa L.), as an ancient crop, has been widely cultivated throughout the world for 2000 years. Oat plays a role in blood glucose lowering (14), weight loss (15), and prevention of cardiovascular diseases (16) and cancer (17). Our previous clinical study has showed that short- and long-term oat intake had significant effects on controlling hyperglycemia, lowering blood lipid, and reducing weight for overweight T2DM patients (14). Furthermore, we applied metabolomics analysis to demonstrate underlying benefits and mechanisms of naked oat bran on serum metabolites of dyslipidemic rats (18). It has been found that oat β glucan (OG), the main functional factor of oat, is not digested in the human small intestine and enters the colon, where it is fermented by the gut microflora (19). The health benefits of OG as soluble dietary fiber have been widely reported, such as regulating blood glucose (20) and improving immune imbalance (21). Importantly, OG has been shown to modulate the composition of gut microflora of humans and animals (22, 23). Given a significant role for gut microbiota in metabolism, it is likely that the effects of OG on the host are multifaceted and involve regulation of microbe-host interactions. Although studies have revealed that OG intake favored the glycaemic control of diabetes mellitus patients, the effect of OG in the control of the occurrence and development of diabetic nephropathy remains poorly understood. However, considering the moderating effects of OG on gut microbiota, it can be speculated that OG might possess immeasurable potential in the treatment of diabetic nephropathy.

Therefore, the aim of the present study was to explore the effects of oat β glucan on delaying diabetic nephropathy progression and whether the effects are caused by intestinal flora through 16S rRNA genes sequencing. This study will provide a scientific basis for prevention and treatment of diabetic nephropathy.

## Materials and Methods

### Materials and Reagents

Common feedstuffs were purchased from Beijing Keaoxieli Feed Co, Ltd (American Institute of Nutrition-1993 Maintenance, AIN-93M). OG (purity 70%) was purchased from Shaanxi Senfu Natural Products Co, Ltd. ELISA microplate reader (MA Model 450 Microplate Reader) was purchased from Labsystems Multiskan Co, Ltd.

### Animal Treatment

Sixty male Sprague Dawley rats (weighing 180–220 g) of specific pathogen free (SPF) class were purchased from the Department of Laboratory Animal Science, Peking University Health Science Center (Beijing, China). Rats were kept under controlled conditions (23 ± 1°C, 55% humidity, 12:12 h of light:darkness alternating) and fed standard chow and water. All experimental procedures were reviewed and approved by the Institutional Animal Care and Use Committee of Peking University (LA2021459) and complied with the Guide for the Care and Use of Laboratory Animals (NIH publication no. 85–23, revised 1996).

After 1 week of acclimatization, DN model was established in 40 rats by left nephrectomy and single intraperitoneal injection of streptozotocin (65 mg/kg·bw). These rats were randomly divided into the model group (distilled water gavage, referred to as the DN group), and three oat β glucan intervention groups [0.275, 0.55, and 1.1 g/kg·bw oat β glucan lavage, hereinafter referred to as the low-dose oat β glucan (LOG), medium-dose oat β glucan (MOG), and high-dose oat β glucan (HOG) group, respectively]. Another 20 rats underwent sham operation and were randomly divided into normal control group (distilled water gavage, NC group) and oat β glucan control group (0.55 g/kg·bw oat β glucan gavage, OGC group). Animals were treated by oral gavage for 8 consecutive weeks. The dose selections for OG were based on our preliminary study (14). We found that daily consumption of naked oat (100 g) exerts significant clinical improvements in diabetes mellitus patients, with the content of functional factor β glucan being about 5%. In clinical practice, the dose of 5 g/d oat β glucan for patients weighing 60 kg is equivalent to 0.55 g/kg·bw for rats, which is set as the MOG group. In addition, the LOG group (0.275 g/kg·bw) and the HOG group (1.100 g/kg·bw) were added.

During the experiment, the general conditions of animals were observed daily, including coat color, mental state and daily activities, and the food intake, body weight, and glucose levels were regularly recorded. Twenty-four-hour urine and fecal samples were collected using metabolic cages, and then stored at −80°C at weeks 0, 4, and 8.

### Blood Glucose, Renal Function, and Early Indexes of Nephropathy Assay

After fasting the rats for 6 h, blood glucose levels were determined weekly using a glucometer. The concentrations of blood urea nitrogen (BUN), serum uric acid (SUA), and serum creatinine (SCr) were measured using automatic biochemical instrument purchased from Japan Olympus Corporation automatic biochemical instrument AU480.

Urine samples were processed according to the instructions of the ELISA kits (E-EL-M0389C, Elascience, China), and the contents of IgG, cystatin C (CysC), and retinol binding protein (RBP) were detected separately. Standards or urine samples (50 μL) were added and incubated at 37°C for 60 min. After washing each well 5 times, TMB chromogen was added in order and incubated at 25°C for 15 min, avoiding light. Then, the reaction was terminated with a stop solution and the optical density (OD) value of each well was measured at a 450 nm wavelength in a microplate reader. After the reaction is terminated at 25°C for 15min, read the OD of each well and convert the sample concentration according to the regression equation of the standard curve. Sample concentration was calculated according to linear regression equations of the standard curve.

### Morphological Observation

The kidney was used for histopathological examination. Kidney samples were fixed in 10% formaldehyde, dehydrated by a gradient mixture of ethyl alcohol and water, and transparentized with xylene. Then samples were embedded with paraffin and sectioned into 5 μm slices. Subsequently, sections of paraffin-embedded kidneys were stained by means of hematoxylin-eosin (HE) staining. The kidney tissue section slides were incubated in hematoxylin for 5 min, washed with tap water, incubated in 95% ethanol, and stained with eosin and phloxine for 1 min. Then, the sections were dehydrated in ethanol and xylene, and sealed with neutral resins. Finally, tissues were examined under a light microscope (Olympus BX43) followed by image acquisition.

### Inflammatory Indicator Assay

Serum samples were processed according to the instructions of the ELISA kit (Invitrogen, Carlsbad, CA, USA), and the contents of Lipopolysaccharides (LPS), interleukin 6 (IL-6), interleukin 8 (IL-8), tumor necrosis factor-α (TNF-α), vascular endothelial growth factor (VEGF), and monocyte chemotactic protein 1 (MCP-1) were detected separately. Antigens were properly diluted with coating buffer; then 5% Bovine Serum Albumin (BSA) was added into each well and incubated at 37°C for 40 min. The plate was then washed 3 times with washing solution, 3 min for each, after which, diluted samples were added to each well at 37° for 40–60 min. The enzyme-labeled antibody was added, incubated at 37°C for 30–60 min, and the substrate was added away from light at 37°C for 3–5 min. Finally, color was developed with TMB substrate for 20 min before stop solution was added to stop the reaction. The concentrations were determined using a microplate reader at a wavelength of 450 nm and calculated based on a standard curve.

Urine samples were processed according to the instructions of the ELISA kit (Invitrogen, Carlsbad, CA), and the contents of interferon γ induced protein 1 (IP-10), MCP-1, and macrophage inflammatory protein-1δ (MIP-1δ) were detected separately. Fifty micro liter of samples were incubated in ELISA wells of microtiter strips coated with the antibody. After four washes, a stabilized TMB chromogen was added and incubated at 25°C for 30 min in the dark. The reaction was stopped and absorbance at 450 nm was measured within 2 h in an ELISA reader. A standard curve was produced using freshly prepared serial dilutions of the kit's reference standard.

### Illumina Sequencing

The gut microbial genomic DNA was extracted from stool samples using a stool DNA extraction kit (QIAamp^®^Fast DNA Stool Mini Kit). The feces samples (200 mg) were put in sterile centrifuge tubes and vortexed for 1 min after adding InhibitEx buffer. The solution was incubated at 70°C for 5 min, centrifuged for 1 min at 3,400 g, and the supernatant removed. The supernatant (200 μL) was then transferred into a sterile microcentrifuge tube, along with 25 μL Proteinase-K. Two hundred micro liter of absolute ethanol was then added and mixed by vortexing. Then, samples were pipetted to the QIAamp spin column and centrifuged for 1 min. Buffer AW1 (500 μL) was added, and the mixture was centrifuged for 3 min. The DNA was eluted in 200 μl of Buffer ATE, directly pipetted on the QIAamp membrane, and collected in a clean Eppendorf tube.

Quality and quantity of the DNA were evaluated using spectrophotometrically with NanoDrop 2000 (Thermo Fisher Scientific, USA), as well as standard 1% agarose gel electrophoresis. The 16S rRNA genes V3–V4 hypervariable region was amplified using primers 341F (CCTACGGGRSGCAGCAG) and 806R (GGACTACVVGGGTATCTAATC). Using the diluted genomic DNA as a template, PCR was performed with KAPA HiFi HotStart ReadyMix PCR kit high-fidelity enzyme (Kapa Biosystems Inc., Boston, MA, USA). All PCR products were detected by 2% agarose gel electrophoresis and recovered by the AxyPrep DNA Gel Extraction Kit (Axygen, Union City, USA). Then library quality was checked using a Thermo NanoDrop 2000 UV microphotometer and 2% agarose gel electrophoresis.

Illumina HiSeq2500 PE250 sequencing platform was performed after the qualified libraries were mixed according to the target data volume. The qualified double-stranded DNA library was transformed into a single-stranded circular DNA library through DNA-denaturation and circularization. Subsequently, the single chain molecules were fixed onto the Solexa Sequencing Chip (flow cell) and amplified by PCR. DNA polymerase I and four types of fluorescently labeled dNTP were added, one base was inserted in each cycle, and the fluorescence signal was captured. Finally, fluorescent groups and terminating groups were chemically cut and a “sticky” end was introduced at the 3′-end of the fragment. The second round of single nucleotide base was incorporated and the fluorescence signal results collected in each round were counted successively to obtain the sequence of template DNA fragment.

Bioinformatics analysis software such as Pandaseq, uSearch, Qiime, and Picrust were used for data Mosaic filtering and species annotation analysis. R language data packet, RDP database, LDA Effect Size (LEfSe), and other methods were used to analyze the classification operating units, species abundance and diversity, and significant differences. *P* < 0.05 was used as the criterion for significance. The correlation between different species and phenotypic indicators and the correlation between species and species were computed in R (version 3.6.2). *R*-values between 0.7 and 1.0 together with *P* < 0.033 can be considered highly correlated.

### Statistical Analysis

Data were analyzed using SPSS software version 27.0. The experimental data are expressed as the mean ± standard deviation (X ± SD). Normality and variance homogeneity of data were analyzed by using a single sample K–S test and Levene's test for homogeneity of variances before analysis. Pairwise comparisons were tested by Kruskal–Wallis non-parametric tests. *P* < 0.05 was used as the criterion for significance.

## Results

### OG Reduced the Blood Glucose Level in DN Rats

**Figure 1:**
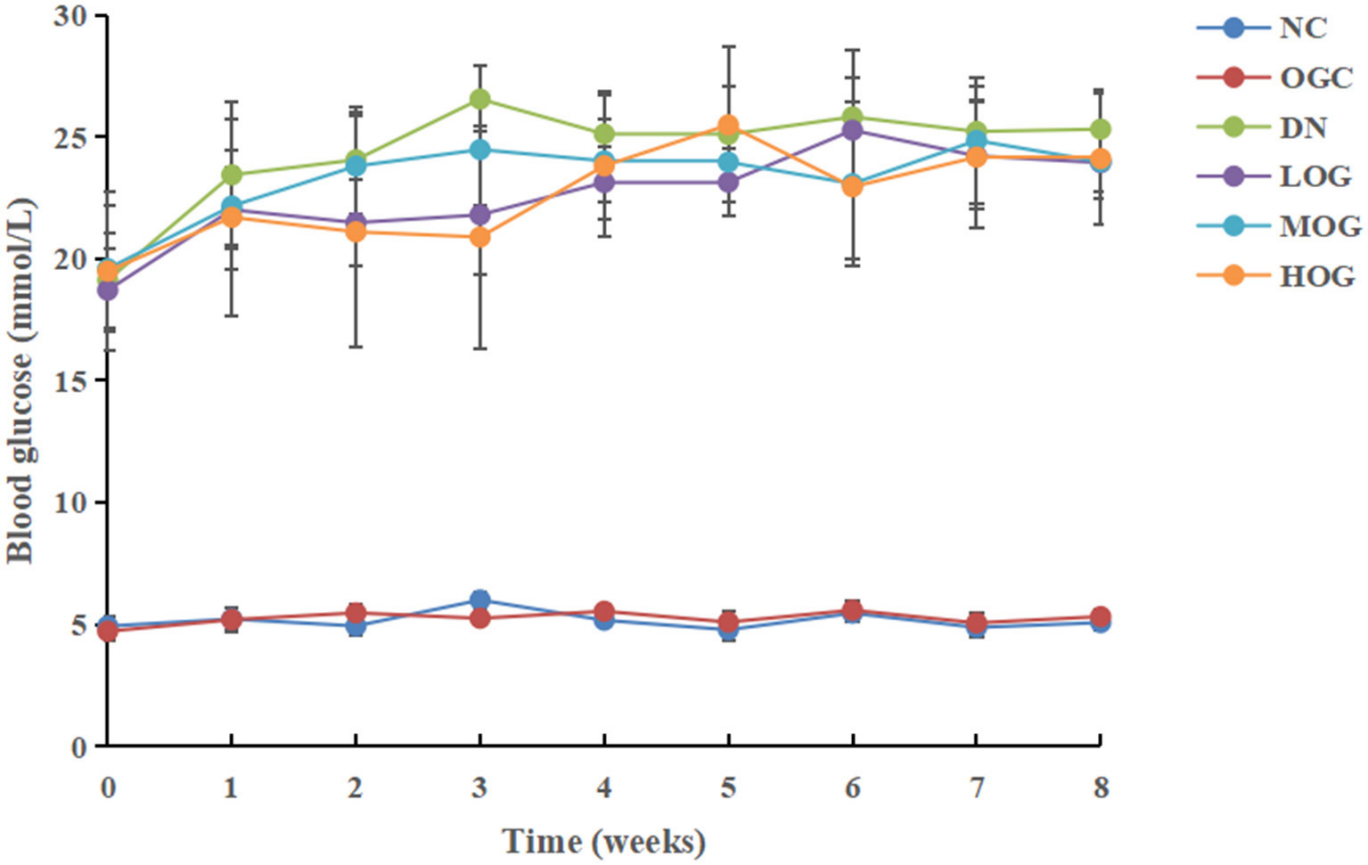
Blood glucose level changes of the rats in each group over time.

At the beginning of the study (week 0), blood glucose levels in the DN group increased significantly compared to the NC group (*P* < 0.05; Figure 1). Blood glucose levels of DN rats gradually increased with time, but no significant changes were observed in the NC group. Compared with the DN group, blood glucose levels decreased significantly in all intervention groups, among which the levels decreased more obviously in the HOG group (*P* < 0.05). There were no significant differences in blood glucose between the NC and OGC groups (*P* > 0.05).

### OG Improved Renal Function in DN Rats

At week 0, compared with the NC group, the BUN, SUA, and SCr levels in the DN group increased significantly (*P* < 0.05), indicating that the model was established successfully. As the intervention time extended, renal function in DN rats had improved by OG to different degrees, but no dose–effect relationship was seen. OG treatment had no effect on the BUN, SUA, and SCr levels, and SUA/SCr ratio in normal rats (Table 1).

**Table 1:** Changes in renal function of the rats in each group (*n* = 10).

**Indicators**	**Groups**	**Week 0**	**Week 4**	**Week 8**
**BUN (mmol/L)**				
	NC	4.54 ± 0.83	4.99 ± 1.14	6.90 ± 1.90
	OGC	4.19 ± 0.42	4.80 ± 0.97	7.10 ± 1.19
	DN	13.04 ± 3.67^&&^	11.15 ± 3.25^&&^	14.80 ± 3.63^&&^
	LOG	12.05 ± 2.60	9.25 ± 1.59^*^	10.39 ± 2.04^**^
	MOG	13.24 ± 3.08	10.70 ± 3.30	12.29 ± 2.50
	HOG	15.40 ± 2.63	10.00 ± 1.26	14.45 ± 3.26
**SUA (umol/L)**				
	NC	48.8 ± 29.85	41.30 ± 16.03	94.20 ± 23.60
	OGC	43.89 ± 13.62	39.00 ± 10.91	101.44 ± 18.66
	DN	105.22 ± 42.9^&&^	151.91 ± 64.14^&&^	181.05 ± 47.87^&&^
	LOG	79.65 ± 24.59	323.62 ± 81.25	250.88 ± 37.29
	MOG	92.29 ± 41.93	138.43 ± 62.20^*^	191.43 ± 46.91
	HOG	74.96 ± 6.42	124.44 ± 46.67^*^	178.56 ± 22.51
**SCr (umol/L)**				
	NC	32.40 ± 3.06	37.70 ± 3.40	33.90 ± 13.89
	OGC	29.56 ± 1.81	42.11 ± 18.31	35.11 ± 10.24
	DN	37.67 ± 2.56^&&^	38.45 ± 4.29	21.34 ± 11.46^&^
	LOG	38.89 ± 3.75	34.68 ± 5.88	17.95 ± 7.41^*^
	MOG	36.14 ± 6.87	32.86 ± 6.85	15.71 ± 9.50^*^
	HOG	39.17 ± 4.09	34.75 ± 6.86	20.80 ± 9.01
**SUA/SCr**				
	NC	1.47 ± 0.80	1.09 ± 0.65	3.28 ± 2.09
	OGC	1.50 ± 0.49	1.02 ± 0.40	3.11 ± 1.01
	DN	2.81 ± 1.18^&&^	4.04 ± 2.08^&&^	28.53 ± 3.22^&&^
	LOG	2.11 ± 0.85	9.83 ± 5.77	15.48 ± 1.76^*^
	MOG	2.55 ± 0.90	4.57 ± 2.58	24.30 ± 2.99
	HOG	1.94 ± 0.34	3.73 ± 1.75^*^	15.39 ± 1.27^*^
**IgG (μg/mL)**				
	NC	10.30 ± 0.65	10.80 ± 0.98	10.99 ± 0.64
	OGC	10.79 ± 0.92	11.20 ± 0.76	10.94 ± 0.84
	DN	10.69 ± 0.72	10.04 ± 1.14	10.73 ± 0.88
	LOG	10.27 ± 0.91	9.40 ± 0.70	9.81 ± 0.54
	MOG	11.55 ± 0.73	10.33 ± 1.01	11.24 ± 0.51
	HOG	10.01 ± 0.72	10.03 ± 0.82	11.17 ± 0.55
**CysC (μg/L)**				
	NC	904.26 ± 56.67	997.84 ± 67.17	1004.63 ± 114.2
	OGC	949.41 ± 115.87	1,054.01 ± 102.01	944.93 ± 104.23
	DN	984.86 ± 116.18	998.6 ± 44.89	955.01 ± 86.10
	LOG	999.04 ± 55.91	1,179.26 ± 113.17	1,011.35 ± 138.12
	MOG	920.30 ± 109.66	1,050.60 ± 161.49	939.34 ± 98.48
	HOG	1,121.05 ± 34.09	1,086.28 ± 133.78	1,037.47 ± 91.06
**RBP (μg/L)**				
	NC	14.05 ± 1.24	14.26 ± 0.78	13.83 ± 1.18
	OGC	15.62 ± 0.93	15.12 ± 1.12	15.41 ± 1.17
	DN	15.68 ± 1.22	16.93 ± 1.22	16.31 ± 0.87
	LOG	16.92 ± 0.93	16.16 ± 0.94	17.05 ± 1.13
	MOG	16.74 ± 1.43	16.38 ± 1.33	17.02 ± 1.27
	HOG	15.74 ± 0.44	14.44 ± 0.90	15.35 ± 1.11

*Values are expressed as the mean ± SD*.

& *P < 0.05*,

&& *P < 0.01, compared with the NC group*;

* *P < 0.05*,

** *P < 0.01, compared with the DN group*.

No statistically significant differences in the IgG, CysC, RBP levels were found among the all groups at any time point (*P* > 0.05), indicating that OG did not affect diagnostic indices for the early detection of DN.

### OG Ameliorated Pathological Changes of the Kidney in DN Rats

**Figure 2:**
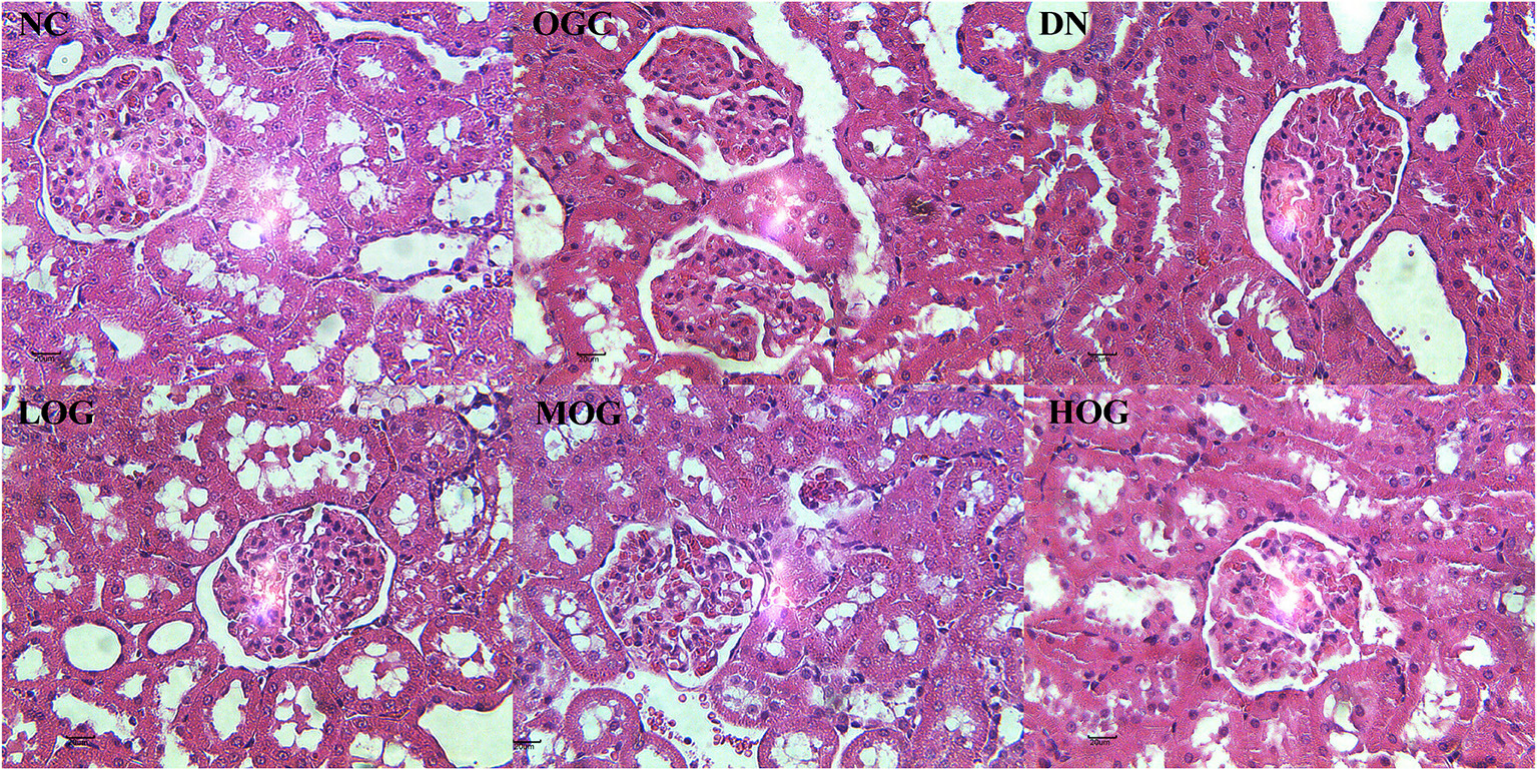
Pathological changes of the kidney of the rats in each group. Scale bar: 20 μm; original magnification 400×.

At week 8, the rats in the NC group showed normal histological features, with neatly arranged renal tubular epithelial cells and normal shape and size of glomeruli. Mesangial hypercellularity and glomerular basement membrane thickening were revealed, renal tubular epithelial cells were disorder and shed, and slight hyaline changes were seen in the DN group. OG treatment resulted in a trend improvement in thickening of the glomerular basement membrane and injury of renal tubular epithelial cells compared with the DN group. The structures of glomeruli and renal tubules were normal, with the epithelial cells arranging neatly in the HOG group, close to that of the NC group. Slight hyaline changes were still present in the LOG group (Figure 2).

### OG Improved Serum Inflammatory Levels in DN Rats

DN rats increased the serum levels of IL-6, VEGF, and MCP-1 (*P* < 0.05), which were significantly attenuated by OG treatment (*P* < 0.05). Moreover, IL-8 level was decreased significantly in the MOG group and LPS level was decreased significantly in the HOG group compared with the DN group (*P* < 0.05; Table 2).

**Table 2:** Serum levels of inflammatory factors of the rats in each group (*n* = 10).

**Groups**	**LPS (u/L)**	**IL-6 (ng/L)**	**IL-8 (ng/L)**	**TNF-α (ng/L)**	**VEGF (ng/L)**	**MCP-1 (ng/L)**
NC	19.42 ± 0.95	131.08 ± 3.49	779.88 ± 45.89	306.32 ± 14.99	279.93 ± 12.52	728.48 ± 38.49
OGC	19.64 ± 0.76	137.15 ± 11.30	795.64 ± 31.64	270.17 ± 12.06	312.54 ± 13.39	715.31 ± 37.14
DN	22.98 ± 1.09	145.96 ± 5.67^**^	756.50 ± 41.92	287.47 ± 16.18	322.66 ± 11.40^**^	825.19 ± 38.24^**^
LOG	20.72 ± 0.87	155.99 ± 9.92	760.87 ± 27.48	280.57 ± 8.94	269.94 ± 16.70^##^	796.75 ± 42.81
MOG	18.18 ± 1.14	144.70 ± 7.90	734.63 ± 41.00^#^	281.01 ± 16.79	322.44 ± 15.91	787.90 ± 25.32
HOG	17.18 ± 1.08^#^	129.71 ± 6.48^#^	759.04 ± 46.77	274.11 ± 18.54	323.90 ± 20.36	762.79 ± 17.79^#^

*Values are expressed as the mean ± SD*.

** *P < 0.01, compared with the NC group*;

# *P < 0.05*,

## *P < 0.01, compared with the DN group*.

No statistically significant differences in urine inflammatory indices were found between the NC and DN groups at any time point (*P* > 0.05). A trend of declining MIP-1δ level over time was observed, but did not reach statistical significance in the MOG group compared with the DN group. Furthermore, no statistically significant differences in urine inflammatory indices were found between the NC and OGC groups (*P* > 0.05; Table 3).

**Table 3:** Inflammatory changes in the urine of the rats in each group (*n* = 10).

**Indicators**	**Groups**	**Week 0**	**Week 4**	**Week 8**
**MIP-1δ** **(ng/L)**				
	NC	546.77 ± 43.53	528.78 ± 43.73	501.62 ± 15.61
	OGC	575.87 ± 43.93	550.00 ± 24.85	525.20 ± 26.12
	DN	543.76 ± 27.23	543.58 ± 33.13	546.77 ± 31.88
	LOG	486.91 ± 42.58	490.12 ± 41.81	506.64 ± 28.29
	MOG	545.10 ± 32.47	537.72 ± 36.21	511.12 ± 39.95
	HOG	505.14 ± 41.49	502.13 ± 46.43	513.82 ± 34.91
**MCP-1 (ng/L)**				
	NC	628.44 ± 66.62	674.38 ± 82.03	618.31 ± 75.78
	OGC	535.57 ± 51.51	612.61 ± 49.49	521.67 ± 29.75
	DN	606.99 ± 67.08	591.47 ± 78.77	600.63 ± 44.96
	LOG	499.75 ± 79.33	574.32 ± 78.21	599.39 ± 81.11
	MOG	736.87 ± 76.23	685.42 ± 65.82	659.34 ± 98.48
	HOG	726.03 ± 66.05	663.57 ± 40.61	636.56 ± 51.72
**IP-10 (ng/L)**				
	NC	111.78 ± 7.54	110.35 ± 7.95	112.33 ± 7.57
	OGC	108.42 ± 9.09	106.64 ± 6.42	105.17 ± 8.17
	DN	105.51 ± 11.21	108.39 ± 10.61	109.05 ± 8.71
	LOG	123.65 ± 9.09	126.64 ± 6.84	123.74 ± 7.00
	MOG	118.09 ± 8.09	109.92 ± 5.83	119.99 ± 11.11
	HOG	110.23 ± 7.71	112.14 ± 9.34	106.40 ± 7.53

### OG Regulated Gut Microbiota Dysbiosis in DN Rats

**Figure 3:**
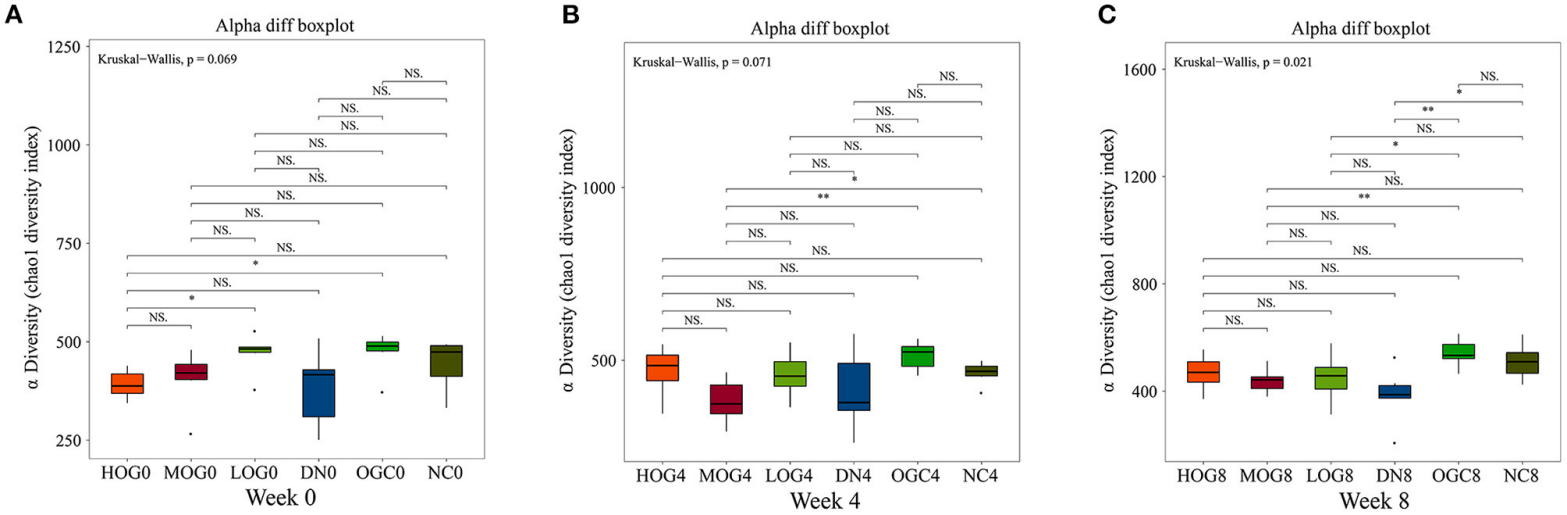
Chao1 index of gut microbiota at 0 **(A)**, 4 **(B)**, and 8 **(C)** weeks. Chao1 index was used to estimate the total number of species in samples: larger Chao1 values indicate more species.

**Figure 4:**
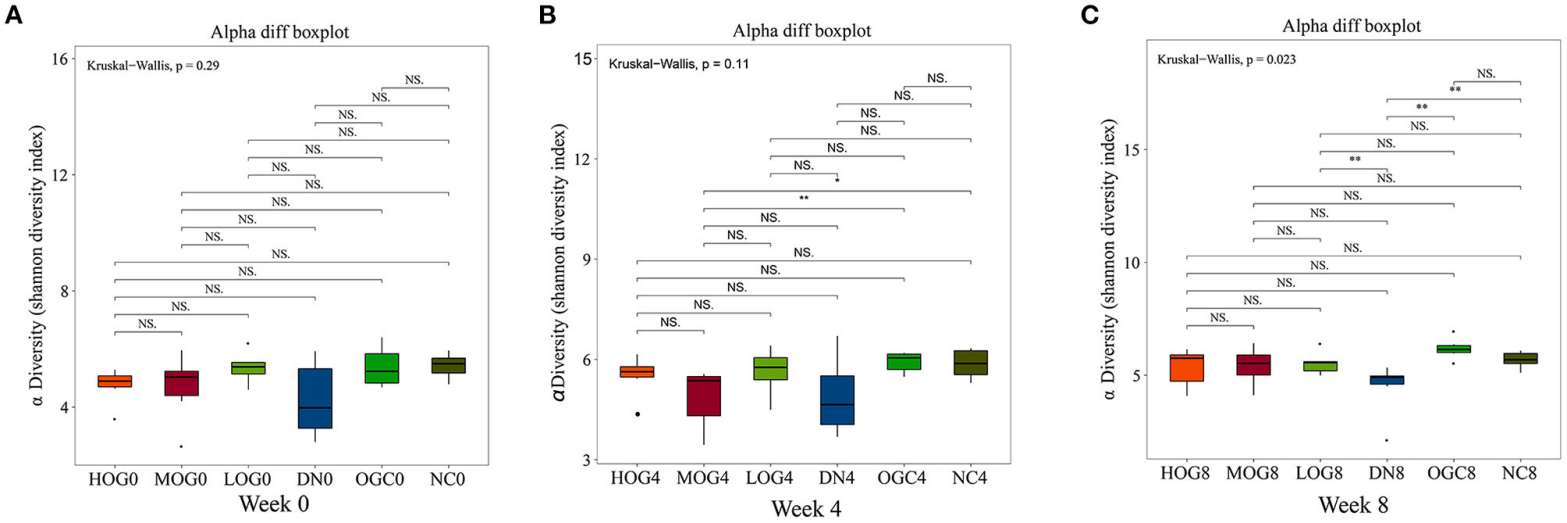
Shannon index of gut microbiota at 0 **(A)**, 4 **(B)**, and 8 **(C)** weeks. Shannon index **(B)** was used to reflect the diversity of the sample population: the larger the Shannon, the higher the diversity of the flora.

The alpha diversity of bacterial communities was evaluated according to Chao1 index and Shannon's diversity index (Figures 3, 4). At week 0 and week 4, there was no statistical significance in Chao1 index and Shannon's diversity index among each group (*P* > 0.05). At week 8, the Chao1 and Shannon's diversity index decreased significantly in the DN group compared with those in the NC group, while the LOG, MOG, and HOG groups showed higher Chao1 and Shannon's diversity index than those of the DN group (*P* < 0.05). Among them, the increase in the HOG group was the most obvious. There was no statistically significant difference between the NC and OGC groups at all three time points (*P* > 0.05).

**Figure 5:**
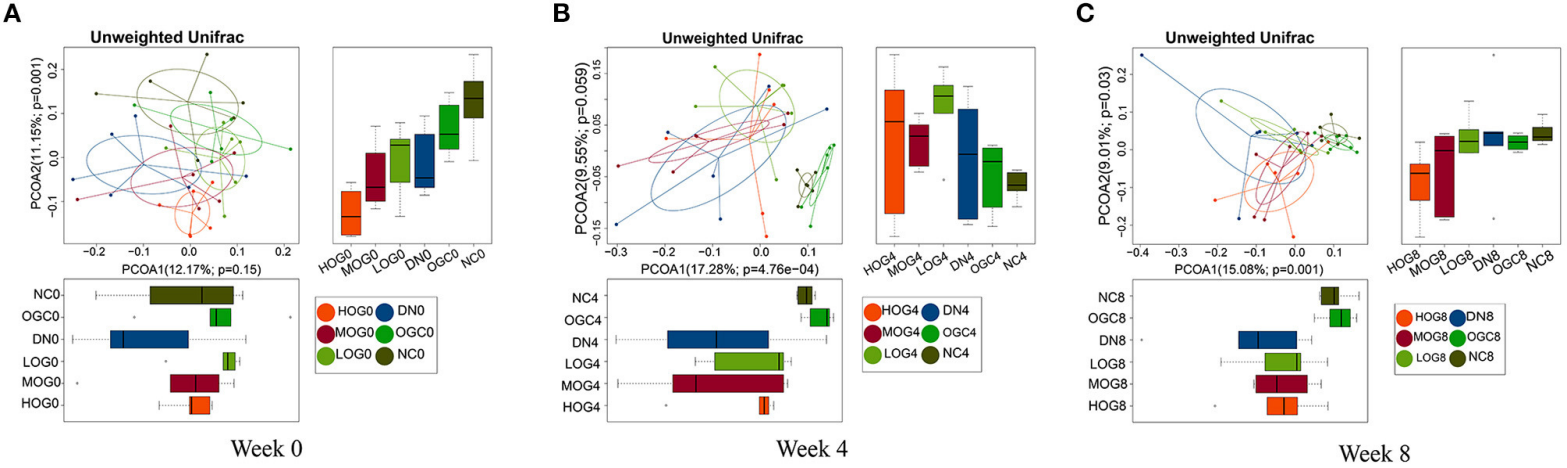
Beta diversity of gut microbiota at 0 **(A)**, 4 **(B)**, and 8 **(C)** weeks. PCoA plots reflect the beta diversity of gut microbiota: the abscissa represents the contribution rate of the first principal component to the sample difference, the ordinate represents the second principal component, and the percentage represents the contribution rate of the second principal component to the sample.

Beta diversity was measured via unweighted Unifrac distance, using the first two principal coordinates to visualize the dissimilarity distances and the variation between samples (Figure 5). The principal coordinate analysis (PCoA) scatterplot illustrated the dissimilarities of gut bacterial communities between the NC and DN groups at both 4 and 8 weeks (*P* < 0.05). Compared with the DN group, the community structures observed were significantly different in the LOG group (*P* < 0.05). However, there was no significance difference among the DN, MOG, and HOG groups. Microbial communities were not statistically different between the NC and OGC groups (*P* > 0.05).

**Figure 6:**
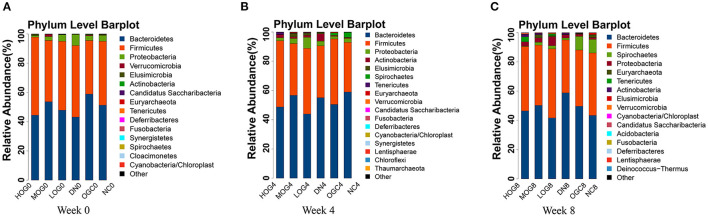
Gut microbial alterations at phylum level of the rats in each group at 0 **(A)**, 4 **(B)**, and 8 **(C)** weeks. Bar plots reflect average relative abundance profiles at phylum level.

**Figure 7:**
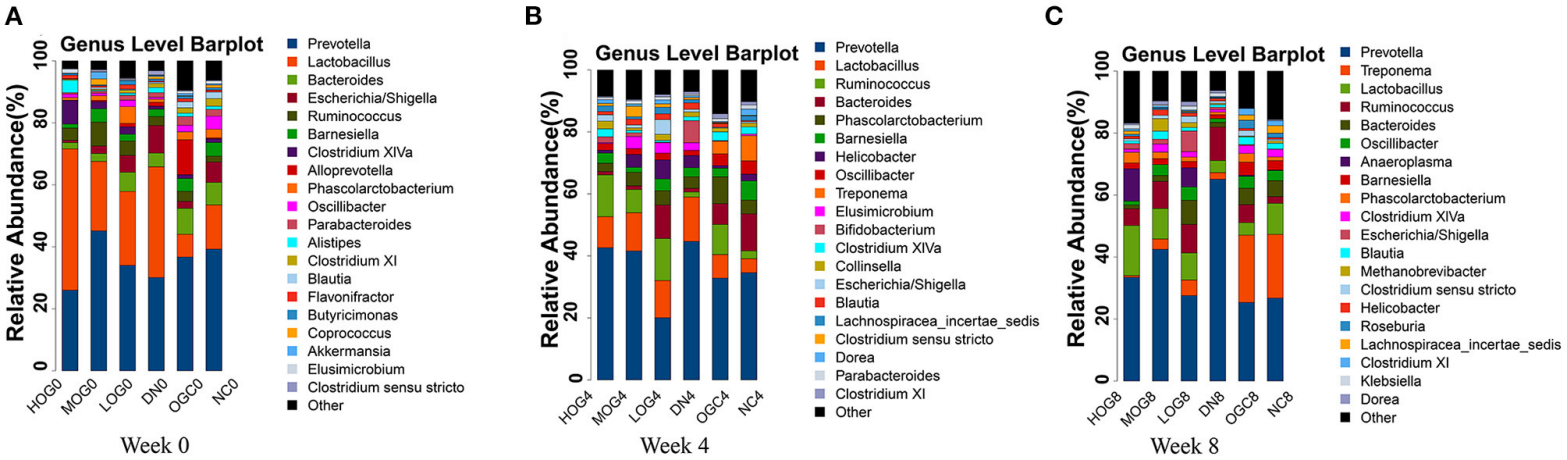
Gut microbial alterations at genus level of the rats in each group at 0 **(A)**, 4 **(B)**, and 8 **(C)** weeks. Bar plots reflect average relative abundance profiles at genus level.

**Figure 8:**
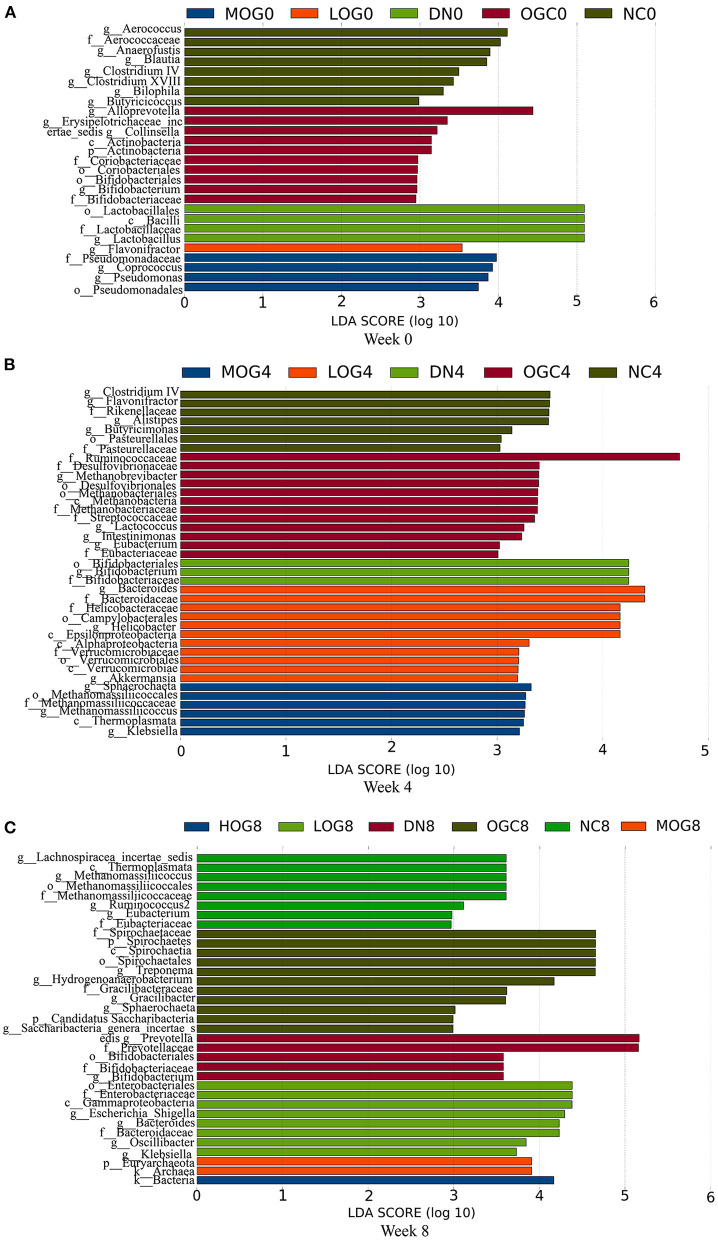
LEfSe significant differences in abundance (LDA score > 2) of the rats in each group at 0 **(A)**, 4 **(B)**, and 8 **(C)** weeks.

The relative abundance at the phylum level of six groups at each time point was shown in Figure 6. The dominant phyla in the gut microbiota were *Firmicutes* and *Bacteroidetes*. With prolonged treatment time, the ratio of *Firmicutes*/*Bacteroidetes* showed a decline in the DN group. The ratio of *Firmicutes*/*Bacteroidetes* increased over time in the MOG and HOG groups, and the elevation in the HOG group is even greater. Figure 7 showed the results for the highest relative abundance ranking top 20 at genus level. Many microbial taxa significantly differed among the groups using LEfSe analysis (Figure 8). There was a significant increase in the relative abundance of *Bifidobacterium* and *Klebsiella*, and a reduction in *Clostridium cluster IV, Butyricicoccus, Eubacterium, Ruminococcus*, and *Spirillum* in the DN group compared with the NC group (*P* < 0.05). Compared with the DN group at week 4, the abundance of *Ruminococcus* was elevated significantly in the LOG group (*P* < 0.05). Compared with the DN group at week 8, the abundance of *Prevotella* was decreased significantly in the LOG group, the abundance of *Butyricicoccus* was increased significantly in the MOG group, and the abundance of *Akkermansia* was increased significantly in the HOG group (*P* < 0.05). In the OGC group, the abundance of *Akkermansia* was significantly increased, and the abundance of *Enterococcus* was significantly decreased (*P* < 0.05).

**Figure 9:**
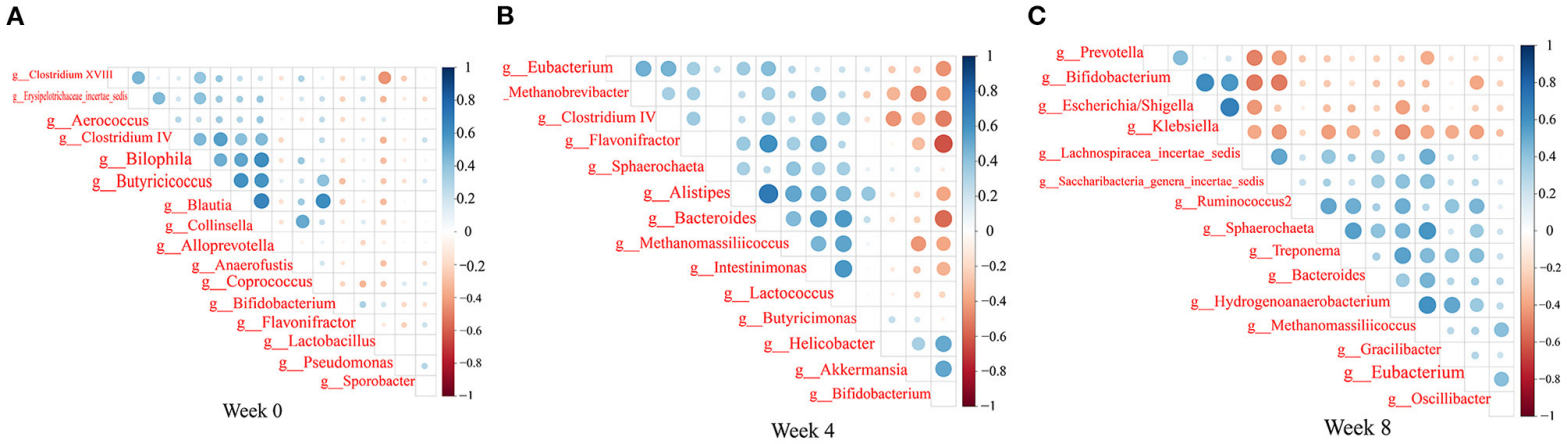
Heatmaps of the correlation coefficients among the differential microorganisms of the rats in each group at 0 **(A)**, 4 **(B)**, and 8 **(C)** weeks. Coloring indicates the direction of association (blue: positive; red: negative). The degree of correlation is highlighted in color: the stronger the correlation, the darker the color.

We observed trends in association among gut microbiota (Figure 9). *Bacteroides* abundance had significant positive correlations with *Bifidobacterium* abundance, but significant negative correlation with *Clostridium cluster IV, Eubacterium*, and *Lactobacillus* abundances. *Bifidobacterium* abundance had significant positive correlations with *Escherichia*/*Shigella* and *Klebsiella* abundances. *Clostridium cluster IV* abundance had significant positive correlations with *Lactobacillus* and *Butyricicoccus* abundances. *Klebsiella* abundance had significant negative correlations with *Eubacterium, Spirillum*, and *Saccharibacteria genera incertae sedis* abundances (*P* < 0.05, *r* > 0.4, or *r* < −0.4).

### Association Between Gut Microbiota and Phenotypes

**Figure 10:**
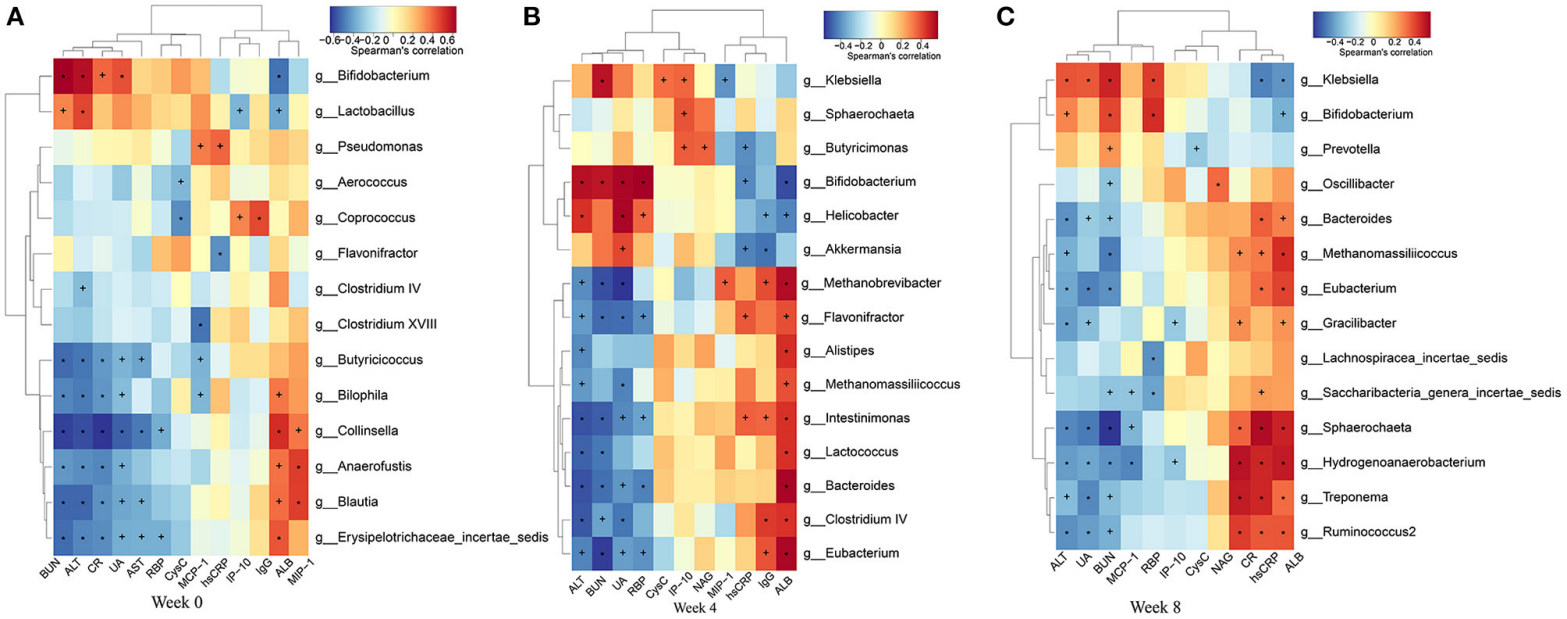
Heatmaps of the correlation coefficients between the phenotypes and the differential microorganisms of the rats in each group at 0 **(A)**, 4 **(B)**, and 8 **(C)** weeks. Coloring indicates the direction of association (red: positive; blue: negative). +*P* < 0.05; *P < 0.01.

To find the main microflora affecting the changes of clinical indicators, we analyzed the correlation between the gut microbiota and phenotypes (Figure 10). BUN showed significant positive correlation with *Bifidobacterium* and *Klebsiella* abundances, and negative correlation with *Eubacterium, Butyricicoccus*, and *Saccharibacteria genera incertae sedis* abundances (*P* < 0.05, *r* > 0.4, or *r* < −0.4). The level of SUA was inversely correlative to *Eubacterium, Ruminococcus, Spirillum*, and *Clostridium cluster IV* abundances, while SUA was significantly positive correlation with *Klebsiella* abundance. SCr showed strong negative correlation with *Butyricicoccus, Ruminococcus, Spirillum*, and *Eubacterium*. MCP-1 was significantly negatively correlated with *Butyricicoccus* abundance. LPS was significantly positively correlated with *Bacteroides* abundance. MIP-1δ was significantly negatively correlated with *Spirillum* and *Saccharibacteria genera incertae sedis* abundances.

### The Altered Function of the Gut Microbiota Was Partly Restored After OG Treatment

**Figure 11:**
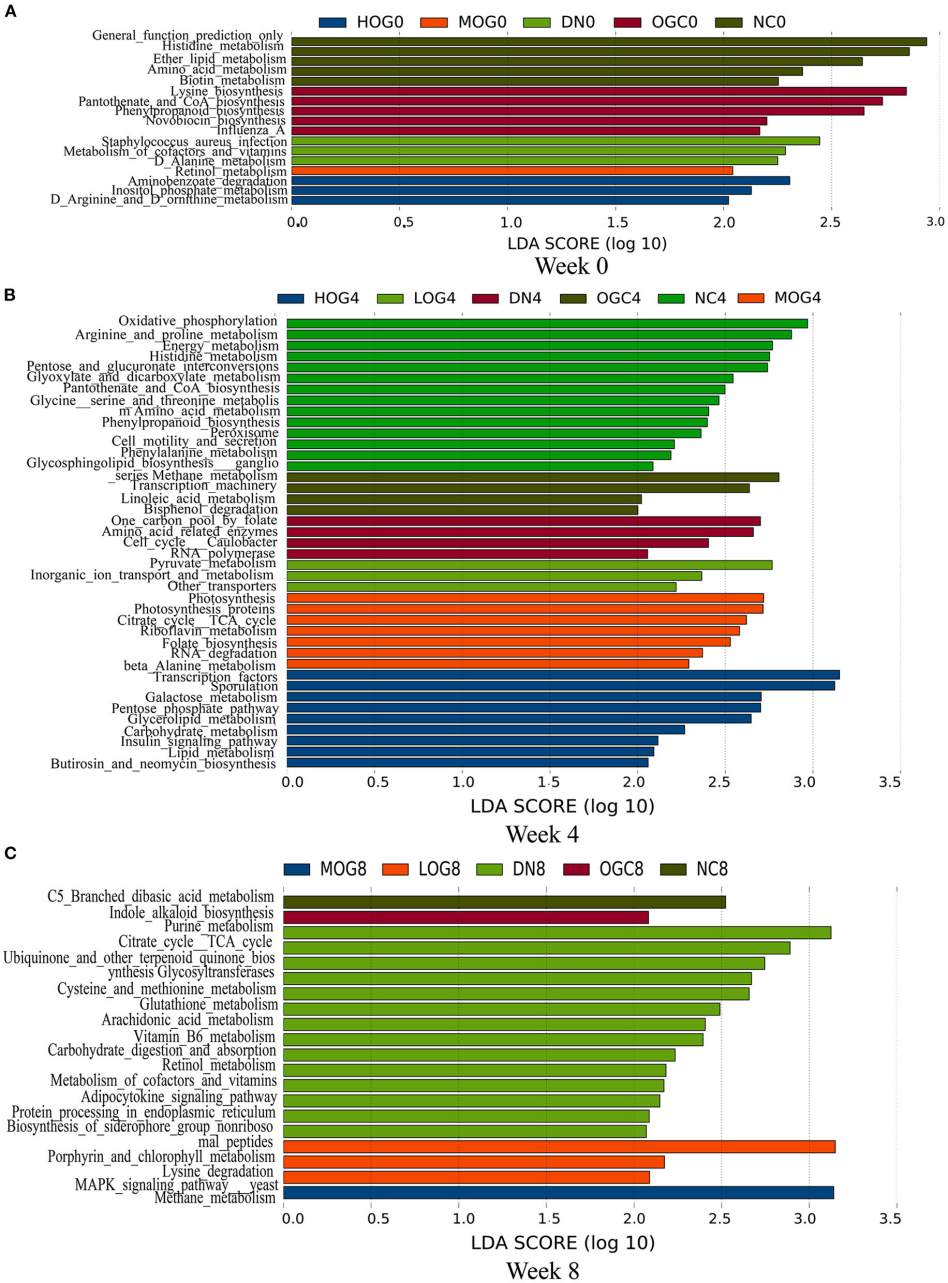
Gut microbial gene functions of the rats in each group at 0 **(A)**, 4 **(B)**, and 8 **(C)** weeks.

To determine how OG affected the metabolism of the intestinal microbiota and hence renal function in DN rats, we performed functional prediction analysis based on the 16S datasets (Figure 11). Amino acid metabolism, retinol metabolism, vitamin B6 metabolism, and purine metabolism pathways were enriched significantly in the DN group. Pyruvate metabolism and lysine degradation pathways enriched in the LOG group. Beta-alanine metabolism, riboflavin metabolism, and citrate cycle (TCA cycle) pathways were significantly enriched in the MOG group. Lipid metabolism and insulin signaling pathways were significantly enriched in the HOG group.

## Discussion

We generated a rat model of DN using left nephrectomy and single intraperitoneal injection of streptozotocin, and explored the effect and mechanism of OG improving renal function by modulating gut microbiota composition in DN rats. We found that OG can regulate changes of intestinal flora, alleviate the inflammatory response, and further delay the development of DN.

Since kidney injury is a progressive process, we measured most of indicators at three time points and observed changes during the process dynamically. In our study, the levels of blood glucose, renal impairment markers, and inflammatory cytokines were increased in DN rats, which is fully consistent with the feature of DN. Elevated blood glucose caused increased levels of oxidative stress, secreted inflammatory cytokines, and caused systemic and local inflammation (24). Accumulating number of researches demonstrated that systemic and renal local inflammation can promote renal tissue damage, which is recognized as crucial factors in DN development. We found that OG treatment could decrease blood glucose level, which is consistent with other studies (25). Of interest, our study revealed for the first time that OG treatment could improve renal function in DN rats, presenting time-effect relationship. Dose-dependent effect was not seen, which possibly owed to the narrow dosage range of OG. Furthermore, OG could also improve the renal morphological changes, objectively reflecting its efficiency in ameliorating renal injury in rats.

Deterioration of renal function shifts the primary site of urea excretion from the kidney to the colon. In turn, the sustained presence of urea in the colon triggers the proliferation of urease-producing bacteria, leading to gut dysbiosis. The shift in microbiota composition enhances gut ammonia production, thus raising the physiological pH of the gut lumen and leading to increased intestinal permeability (26). In our study, elevated LPS levels were presumably due to intestinal flora disturbance and increased gut permeability in DN rats. Consequently, LPS and bacterial products translocated into the circulation and induced both local inflammation and chronic systemic inflammation, further exacerbating the deterioration of renal function. Interestingly, our study found that OG effectively attenuated serum inflammation. This might be attributed to the fact that OG was effective in modulating gut microbiota.

Next, we further explored the changes of the intestinal flora using 16S rRNA genes sequencing and defined whether the changes were a part of the upstream regulatory mechanism in the occurrence and development of DN. In our study, the intestinal flora diversity of DN rats was lower than that of normal rats, which was similar to previous studies (27). OG treatment for 8 weeks induced a positive effect on increasing the diversity of the flora, indicating that OG for a long-term intervention could regulate gut microbiota to maintain intestinal flora homeostasis. Of note, an decreased ratio of *Firmicutes*/*Bacteroidetes* may be a feature of DN-driven disruptions in microbiota, which is consistent with some studies (28, 29). Moreover, the abundance of *Bacteroides* was elevated and butyrate-producing bacteria such as *Butyricicoccus, Eubacterium*, and *Ruminococcus* abundances were reduced in DN rats. Fortunately, supplementation with OG did prevent these changes. *Bacteroidetes* and *Bacteroides* can synthesize LPS and promote neutrophils and monocytes/macrophages activation (30), which might be one of the causes of systemic and local low-grade inflammation. However, *Firmicutes* and butyrate-producing bacteria contribute to short-chain fatty acids (SCFAs) synthesis, maintain the intestinal barrier (31), and regulate the inflammatory reaction (32). We speculated that OG ameliorating renal injury may be partly attributed to modulation of gut microbiota as well as influencing production of gut microbiota-derived SCFAs.

In addition, we also found that relative abundance of *Escherichia*/*Shigella* genus was increased in the DN group, but decreased by OG treatment. Studies have revealed that the increase in *Escherichia*/*Shigella* population may exacerbate gut leakiness by penetrating the intestinal epithelial barrier and produce ethanol by substrate fermentation. Ethanol enters the liver through blood circulation, leading to a disorder of fatty acid metabolism (33). *Escherichia* damages renal tubular epithelial cells and glomerular endothelial cells by producing *Shigella*-like toxins (34). Therefore, the decrease in the abundance of *Escherichia*/*Shigella* by OG treatment may be another reason for improvements in renal function.

We found that *Klebsiella* was increased in DN rats, which was in accordance with several studies (35). Interestingly, we found that *Bifidobacterium* was also increased in DN rats, which was inconsistent with a number of studies. This might be because dysbiotic expansion of *Klebsiella* and other potentially pathogenic bacteria caused the increase of toxins, exfoliation of epithelial cells, and accumulation of mucins in the gut. At this point, *Bifidobacterium* failed to adhere to epithelial cells and colonize the gut, and thus held the dominant position in the feces (36). This may suggest that a longer intervention are needed to implement the expected results.

To probe whether improvement in renal function is due to a direct effect on the changes in gut microbiota by OG treatment or to an effect on blood glucose via modulating gut microbiota, we performed correlation analysis between gut microbiota and phenotypes. We found that *Eubacterium, Butyricicoccus*, and *Ruminococcus* were significantly negatively associated with renal impairment markers, such as BUN, SCr, and SUA. It presents the evidence that these microfloras can regulate the function of the kidney directly. Of course, this requires our further verification and exploration. Furthermore, we also found that *Klebsiella* and MIP-1δ was significantly negatively associated with *Spirillum* and *Saccharibacteria* genera incertae sedis. Although functional roles of the two bacteria remain poorly characterized, there are intertrophic relationships and commensal networks among bacteria in gut. We therefore speculated that both bacteria may have potential probiotic functions, leading to the synergistic decrease of MIP-1δ concentration by OG glycolysis. Further research can be done to clarify this question in the future.

Intestinal flora directly involved in the metabolisms of proteins, amino acids, and carbohydrates (37). Functional annotation analysis showed that disturbed nutrient metabolism existed in DN rats. At week 4, the HOG group enriched microbial gene functions associated with insulin signaling pathway. This might be because the beneficial bacteria that produce SCFAs were dominant in the gut of rats by HOG treatment, such as *Prevotella, Lactobacillus*, and *Ruminococcus*. SCFAs are the main products of the fermentation of non-digestible carbohydrates by the gut microbiome, which can serve as energy source for epithelial cells in the gut and protect the gut barrier (35). This further confirms our above conjecture about the reason for increased *Bifidobacterium* in the feces of DN rats. SCFAs can also improve insulin sensitivity by suppressing chronic inflammation in the host (38). Studies indicated that SCFAs can improve glucose tolerance and attenuate β cell apoptosis in obese and diabetic animals, probably due to the role as a histone deacetylase inhibitor (39). Therefore, we surmised that adequate OG should be introduced as prevention intervention in early-stage DN, ameliorating DN via improving intestinal bacterial metabolism.

In summary, we found that prolonged OG treatment could increase the diversity of the flora, promote the growth of *Lactobacillus* and the release of butyrate, which could ameliorate inflammation and consequently improve renal function in DN rats using 16S rRNA genes sequencing. Our findings provide a safe and effective means for preventing and treating DN. Meanwhile, future studies may opt for a higher purity of oat β glucan.

## Data Availability Statement

The datasets presented in this study can be found in online repositories. The names of the repository/repositories and accession number(s) can be found at: https://www.ncbi.nlm.nih.gov; SUB 11084838.

## Ethics Statement

The animal study was reviewed and approved by the Institutional Animal Care and Use Committee of Peking University.

## Author Contributions

RW designed the experiment and wrote the manuscript. RW, SA, CY, MH, and XH carried out the experiment. ZZ and HQ revised the manuscript. All authors contributed to the article and approved the submitted version.

## Funding

This research was supported by the National Natural Science Foundation of China (Grant No. 81472970).

## Conflict of Interest

The authors declare that the research was conducted in the absence of any commercial or financial relationships that could be construed as a potential conflict of interest.

## Publisher's Note

All claims expressed in this article are solely those of the authors and do not necessarily represent those of their affiliated organizations, or those of the publisher, the editors and the reviewers. Any product that may be evaluated in this article, or claim that may be made by its manufacturer, is not guaranteed or endorsed by the publisher.
